# Cadmium, Lead, Chronic Physiological Stress and Endometrial Cancer: How Environmental Policy Can Alter the Exposure of At-Risk Women in the United States

**DOI:** 10.3390/healthcare11091278

**Published:** 2023-04-29

**Authors:** Elizabeth Olarewaju, Emmanuel Obeng-Gyasi

**Affiliations:** 1 Department of Built Environment, North Carolina A&T State University, Greensboro, NC 27411, USA; 2 Environmental Health and Disease Laboratory, North Carolina A&T State University, Greensboro, NC 27411, USA

**Keywords:** stressor, metals, policy, endometrial cancer, women

## Abstract

The health and life outcomes of individuals are intertwined with the context in which they grow and live. The totality of exposures one experiences affects health in the short term and throughout the life course. Environmental exposure to multiple contaminants can increase stress levels in individuals and neighborhoods with psychosocial stressors such as crime, drug and alcohol misuse, and violence also taking a toll on individual and neighborhood wellbeing. In addition, the availability, organization, and quality of local institutions and infrastructure all affect health in the short and long term. The role of these factors in endometrial cancer will be explored in this paper. In addition, policy implications regarding lead, chronic physiological stress, and endometrial cancer will be explored to ascertain the impact of these factors on at-risk women.

## 1. Introduction

### 1.1. Endometrial Cancer: A Disease of Public Heath Importance

Endometrial cancer (EC)—a tumor that develops from the cells of the inner lining of the uterus [1]—is the most common gynecological cancer [2] and the fourth most common cancer among women in Europe and North America [3]. EC occurs when the cells of the endometrium begin to grow or multiply excessively leading to some parts of the uterine lining becoming thicker and combine to form a cancerous mass of tissues. EC has been categorized into two main pathogenic types [1,4]: Type I is estrogen dependent [5], commonly linked to being obese [6] although with a good survival prospect [3]; Type II is less estrogen dependent and is uncommon as it accounts for 10% of all EC case incidence [7], but it is more malignant with poor prognosis [4] and makes for 40% of deaths in EC patients [8] when compared to Type 1. Although at diagnosis up to 90% of EC cases present irregular bleeding as a major symptom which enables early-stage detection of the disease [9] as a screening method for EC, a multi-disciplinary approach is necessary in the assessment, diagnosis, staging and care of EC patients due to the complexity of the process. EC presents predominantly in postmenopausal women [10], with 417,00 cases and 97,000 fatalities recorded in 2020 [11]. The American Cancer Society opines that women above 65 years should be educated on the risks, predisposing factors and symptoms of EC while ensuring regular screening in the occurrence of any of the symptoms [12]. This is critical as the average EC age–incidence curve indicates that the majority of cases are detected postmenopausally [10], with the peak prevalence happening around the seventh decade of life [13].

EC has a significantly growing prevalence associated with excess weight gain and aging [14] as epidemiological research has revealed that an excess of body weight is responsible for > or = 40% of its prevalence [15], and countries within North America and Europe have a ten times higher rate of occurrence in contrast to less advanced countries [13]. Although the exact cause of EC is not known [2], the expected rise in the incidence rates [16] may be influenced by a combination of obesity [17], diabetes mellitus and cadmium exposure [18], which are major risk factors. The development of EC can be influenced by a variety of factors and a multidisciplinary evaluation of these factors is helpful in predicting the extent of risk that an individual is predisposed to. For example, an individual who has a history of diabetes, obesity and other conditions that predispose to EC is at a higher risk of developing EC. Preventive measures which include healthy lifestyle routines such as physical exercise; weight loss; reduced consumption of fatty foods; no smoking; consumption of fruits, vegetables and vitamins; and clinical supervision of diabetes, obesity, and hormonal therapy [19] can influence the life course of at-risk individuals and reduce EC incidence.

Although surgery remains the primary treatment option for EC [20], a multidisciplinary team approach, comprising gynecologists, oncologists, pathologists and radiologists, among others, is necessary for comprehensive assessment and care of EC patients [21]. Additionally, centralized care minimizes the learning curve for advanced surgical intervention and the number of cases that each surgeon must handle individually, which encourages innovation and solves the learning curve issues that low-volume facilities experience [22]. Clinicians can offer holistic and individualized care to people with EC, increasing their quality of life and prognosis by working collaboratively and leveraging the most recent evidence-based procedures and technologies.

### 1.2. Cadmium—A Heavy Metal of Public Health Importance

Cadmium (Cd) poses a significant environmental and occupational risk, and its derivative compounds have been categorized as poisonous by several regulatory bodies [23], and malignant growths in humans and animals have been traced to its presence in the body [24]. The carcinogenic [25], bio-accumulative, non-essential metal has a number of adverse impacts on human health, including the risk of endometrial, breast, lung and pancreatic cancers as well as abnormalities in calcium homeostasis and renal impairment [10,26]. Cd is a pollutant found in the majority of human meals due to its high rates of soil-to-plant transfer [27], making nutrition the main source of exposure for populations that do not smoke and are not exposed to it at work [28,29]. Human endometrial tissue can accumulate Cd, and women who have a smoking history typically have higher Cd levels [30] because Cd easily builds up in tobacco plants. Smoking is a predominant predictor of Cd exposure which increases blood and kidney Cd levels by two to three times and by four to five times, respectively, in smokers compared to non-smokers [31]. Since food and cigarette smoke are the main sources of Cd compounds, individuals are exposed to them both at the workplace and at home, implying that both occupational and residential environments are contributors to Cd exposure.

#### Cadmium and Endometrial Cancer

Although existing data is inadequate, particularly for EC, hormone-dependent cancers may be more prone to estrogen-mimicking substances such as Cd [10]. Recent studies have indicated that Cd causes adipose tissue malfunction and insulin resistance causing elevated risk of obesity which is a predisposing factor to EC [16]. Exogenous estrogen exposure, such as from plant estrogens or hormone-based medications, can also increase the risk of EC [14]. Cd, among other metals, has a tendency to accumulate in the reproductive organs [32].Various mechanisms have been identified that may connect Cd with the development of cancer, such as oxidative stress, inflammation [33,34], interference with DNA repair and changes in DNA methylation [35,36]. 

### 1.3. Lead—A Heavy Metal of Public Health Significance

Lead (Pb) is one of the toxic metals and a prevalent environmental contaminant which has no proven benefits to human health [24]. Pb builds up gradually in the body over a long time with a half-life of 1 month in blood and three decades in bone and has been associated with chronic stress [37]. Among several other factors, age, lifestyle, place of residence and work all play significant roles in determining the amount of Pb present in the body [30], with exposure mediums being lead-contaminated water distribution systems, gasoline, cosmetics, electronic waste recycling and a host of others as Pb is largely absorbed into the bloodstream through breathing and ingestion [24]. Increased prevalence of autoimmune illnesses, infectious diseases, and cancer may be attributed to metal toxicants that impair the immune system [38] with the risk of developing allergies [39], breast cancer, menstrual abnormalities, pregnancy loss, preterm births and stillbirths being potential outcomes of long-term exposure to heavy metals [24]. Increasing levels of toxin exposure elevate the risk of infertility in women, which is compounded by the hormonal imbalance caused by heavy metal toxicity [40].

Pb actively moves through the blood stream [41] and causes harmful effects in the body. Blood lead level (BLL), which is a time-integrated metric of both historic and recent exposure used in the United States, measures the concentration of Pb present in the blood in micrograms of Pb per deciliter of blood (µg/dL). In situations of persistent and continuous exposure to Pb, the degree of “biologically active” form of Pb in a person’s body is indicated by their BLL [42]. Although, data from recent research studies indicate that bone lead is a more prominent marker of human exposure to Pb as it accurately measures exposures to low levels of Pb contaminants over a long period, it is largely unsuitable for use in extensive population studies [43,44,45]. Over the past five decades, since the first official blood lead surveillance was carried out, there has been a decline in BLLs [46] from a geometric mean of 12.8 µg/dL in the 1970s to about 0.82 µg/dL in 2015–2016 [47], which has been associated with the enactment of federal regulations and control measures in the restricted use of leaded gasoline [48], lead-based paints and lead-soldered food cans [44,49].

Pb is still widely used in consumer products and discharged into the air through the combustion of fossil fuels and oils, the incineration of garbage and fugitive emissions during mineral extraction and smelting despite increasing evidence of its adverse health hazards [50], and even in regions where the control of Pb and its presence has been thorough, there are still significant concentrations in the dust, paint and soil.

#### Lead and Endometrial Cancer

Pb, like Cd, may accumulate in endometrial tissue [32]. Past research studies have attributed Pb poisoning and long-term Pb exposure to oxidative stress [37,51], which is an imbalance between the synthesis and retention of oxygen reactive species (ROS) in tissues and cells, and the physiological ability of the human body to eliminate these reactive substances [51,52,53]. An increase in sensitivity of genetic composition due to oxidative stress can lead to elevated estrogen levels [54], which are a significant predictor of EC. Furthermore, exposure to Pb may elevate the probability of enduring DNA damage through impeding the repair mechanism of DNA or through interacting with proteins responsible for DNA repair [55]. Pb can alter the immune system’s cellular responses by lowering immunoglobulin synthesis, exposing people to more inflammatory disorders [40] and chronic inflammation which may induce development of tumors [56], impair cognitive functions due to impact on the central nervous system [46] and cause osteoporosis [57] and lung cancer [58].

### 1.4. Impact of Pollutants on Immune System

Environmental and occupational exposure to a variety of substances such as heavy metals, chemical wastes, and other toxic materials can increase the risk of developing various diseases. The alteration of the immune system represents a risk factor for the development of tumors, including EC, and, depending on the degree and intensity of Pb exposure, individuals may experience a wide range of biological impacts which are harmful to their neurological, reproductive, cardiovascular and hematological systems [39].

In a study by Zhao et al., individuals with high BLLs in rural China had considerably more cytotoxic T cells (CD3^+^CD8^+^) and fewer Th cells (CD3^+^CD4^+^) than controls [59]. Li et al. identified a substantial drop in the ratio of CD4+/CD8+ (a measure of immune system status) in individuals with high BLLs compared to controls, and there is a negative correlation between the proportion of CD4^+^ T cells and BLLs (r = 0.462, *p* = 0.01) [60]. Individuals in the Pb exposure group had median lymphocyte counts that were lower than those in the control group, according to Zhang et al. [61]. Cao et al. found that BLLs in study participants were linked positively with the proportion of CD4+ central memory T cells (r = 0.312, *p* = 0.01) and negatively with the proportion of CD4+-naive T cells (r = 0.317, *p* = 0.01) [62].

There is mounting evidence that Pb harms innate immune cells as well. In a study by Wells and colleagues, participants had an increase in eosinophils of 4.9% (95% CI: 2.3, 7.6) for every 1 g/dL increase in BLLs [63]. According to several studies by Zhang et al., Pb exposure in individuals was associated with a considerable rise in the quantity of neutrophils, monocytes, eosinophils and basophils as well as the percentage of monocytes [64]. Numerous studies have provided evidence that being exposed to Pb can impact the generation of T and B lymphocytes as well as NK cells, which play a critical role in combating cancer [65]. Kasten-Jolly et al. [66] proposed that the increased levels of reactive oxygen species (ROS) triggered by Pb exposure in females could be linked to higher estrogen levels, as estrogen receptors can prompt various cellular populations in the human body, including immune cells. This may have implications for EC as the immune system plays a crucial role in combating the disease.

These studies when taken together reflect the profound effects metals such as Pb may have on the immune system. In addition, they demonstrate that exposure to Cd and Pb can have harmful effects on the immune system and hormonal function, which could ultimately lead to the development of EC and other health problems.

### 1.5. Endometrial Cancer Risk and Chronic Stress

Cancer risk factors, such as environmental toxicants, have long been postulated to include the increased “wear and tear” on the body driven by prolonged stress [67] and by inhibiting the production and absorption of estrogens which reduce the uterus’ sensitivity to estrogen stimulation, and psychological stress may increase the likelihood of endometrial cancer [68].

The Allostatic Load (AL), an index of chronic stress, is a multi-system, multi-dimensional composite measure that typically includes cardiovascular, metabolic, immunological, and neuroendocrine components and has been shown to be a suitable measure of the adverse consequences of prolonged stress levels on health [69]. Compared to many other stress evaluation metrics, AL considers the biological impact of cumulative stress and takes into consideration people’s responses and reactions to the stress burden [67]. Numerous negative effects can result from prolonged exposure to stress, including sleep disturbances and insomnia [70], endocrine abnormalities [71] and alterations in the immune system [72], leading to an increased tendency of tumor production [73] as the immune system has a specific function in predisposing the initiation and advancement of tumor growth through cancer-promoting inflammation [74]. Through a variety of processes, including DNA damage, increased p53 degradation and control of the tumor microenvironment, stress hormones are bound to cause and advance the growth of cancerous cells [72].

### 1.6. Endometrial Cancer and the Pathogenetic Mechanisms Driven by Metals and Stress

Cadmium affects estrogen signaling, causing oxidative stress, DNA methylation changes and inflammation that are important for the oncogenic process of hormone-dependent tumors. Additionally, it may obstruct the clotting and fibrinolysis processes which may play a role in the carcinogenic process [36,75].

In assessing the mechanisms by which stress may increase the risk of EC, studies which examine cortisol and EC are critical and insightful. Research has found that the risk of endometrial cancer rises with a standard deviation increase in genetically predicted plasma cortisol levels during stress. These findings suggest that high levels of plasma cortisol may raise the risk of endometrial cancer. The connection seems to be higher for the hormonally driven endometrioid histological subtype of endometrial cancer than for the non-endometrioid histological subtype. Furthermore, the levels of estrogen and cortisol are complexly influenced by one another [76,77]. An experimental investigation revealed that endometrioid-type endometrial tumors with high estrogen receptor expression were related with expression of the cortisol and other glucocorticoid receptors and had a bad prognosis [78].

### 1.7. Lead Policy in the United States

In the United States, Pb is one of the most significant environmental health risks, and there are increasing concerns regarding health hazards at the levels of exposure that were previously deemed safe [79], even with a drastic decline in public and local use over the past decades. Several pieces of Pb-related legislation have been passed by the US Congress, regulating Pb levels for commercial and residential purposes in paint, dust and soil, water and the handling of lead wastes [47] owing to substantial evidence from scientific research which has found that the even low levels of Pb exposure are still largely unsafe for human health. Although it is impossible to completely prevent or eliminate Pb, it is possible to reduce exposure to Pb through monitoring Pb levels in environmental biological media and effective regulatory actions.

The US Food and Drug Administration has made reducing dietary Pb exposure a public health goal [80], having designated several action levels to address Pb concentration in packaged foods, beauty products and bottled water combined with an outright ban on the utilization of food cans soldered with Pb in the United States [44]. The Environmental Protection Agency, which is saddled with the responsibility of protecting human health and the environment in the United States, administers Pb control regulations which are aimed at putting the concentration levels of Pb in various mediums of exposure to check. These regulations include the Residential Lead-Based Paint Hazard Reduction Act of 1992 (Title X) as amended through April 2005, the Clean Water Act (CWA), the Toxic Substances Control Act (1976), the Clean Air Act (1963) with changes enacted in 1990, the Safe Drinking Water Act (1974) with current amendments as Title XIV of the Public Health Service Act: Safety of Public Water Systems 2019, the Resource Conservation and Recovery Act (RCRA)—Title 40 CFR parts 260 through 273 regulating management of hazardous waste and the Comprehensive Environmental Response, Compensation, and Liability Act (1980) with its provisions enforcement strengthened by the Superfund Amendments and Reauthorization Act (1986), to mention a few.

These regulations were enacted to guard individuals and families in the United States against Pb exposure as a public health priority with Part 35 of the Title X authorizing the HUD and EPA to demand disclosure of adequate information on hazardous effects of lead-based paints prior to the sale or lease of houses developed before 1978 [81]. Additionally, the CWA as amended in 1972 prohibits discharge of contaminants with Pb inclusive into waterways and regulates industrial waste discharge and prescribed standards for sewer systems [82], and the SWDA, which applies to all public water systems with the aim of enhancing the safety of water which the general public consumes, aims to optimize the nation’s water supply to ensure safe, public consumption [79,83], while Title 40 of the Lead and Copper Rule monitors the exposure of individuals to Pb through drinking water while safeguarding public health and ensuring that Pb concentration levels do not exceed 0.015 µg/L [84,85]. The CAA regulates the amount of Pb that can be in outdoor air, restricting pollutant emissions from industries and was responsible for the final ban of leaded gasoline following a couple of amendments [83]. Prior to the restricted use of Pb in paints, solder and gasoline, the environmental levels of Pb in the United States were higher, implying that older people born before the enactment of the regulations accumulated more Pb during their childhood [47].

Table 1 summarizes the sources of Pb exposure being monitored by some of the Pb regulations highlighted above.

It is important to state that Pb exposure remains a continuing health concern in the US and even globally despite the presence of policies aimed at mitigating the risks of exposure. This may largely be due to most of these policies being focused on remedying post-exposure impacts rather than preventing exposure in the first place or the failure of these policies to take into consideration the cumulative effects of exposure to numerous sources of Pb over a period of time [83]. There is also the need for novel and updated regulations as the current regulations, some of which date to over 30 years ago, may not address present day concerns on the use and exposure of individuals in the US to Pb, even in the lowest concentrations.

### 1.8. Cadmium Policy in the United States

People are exposed to Cd compounds as primary sources (food and cigarette use), suggesting that both occupational and residential environments can influence a person’s exposure to Cd through these sources [31]. Secondary sources of exposure could be mining and smelting of cadmium-containing ores and manufacturing processes involving Cd such as painting, welding and soldering. In the light of increasing data from scientific research on the toxicity of Cd, there has been demand for national and global agencies to put in efforts into regulating its presence in the environment and exposure rates. There is a substantial database to aid the formulation of regulations on exposure in the workplace, health, and environmental levels because the effects of Cd’s toxicity on health are widely known [86]. The Occupational Safety and Health Administration, in ensuring the safety of employees in certain industrial sector, prescribes a permissible exposure limit (PEL) of 5 µg/m^3^ for fumes, dust and cadmium compounds; an action level of 2.5 µg/m^3^ calculated as an 8 h time-weighted average for workplace exposure; and the Separate Engineering Control Air Limit of 15 µg/m^3^ or 50 µg/m^3^ [87], depending on the engineering process being carried out in workplaces where the PEL is not achievable. The prevalence of cigarette smoking and tobacco consumption in the U. S. have significantly decreased as a result of tobacco-control regulations in past decades [88] which may have had impacts on reducing Cd exposure in the US population as numerous states and provinces have adopted comprehensive smoke-free regulations [89].

### 1.9. Effects of U.S. Law and Policy on Stress

In comparison to their more privileged peers, marginalized and vulnerable people who are discriminated against on the basis of gender, social status, ethnicity or race are more prone to experiencing chronic stress [90]. To combat disparities in human and environmental health, there is the need to acknowledge the role of stress in the buildup of such disparities and review the social norms and systems that contribute to incidences of stress. Policies to reduce health disparities include restructuring healthcare systems to be more accessible and inclusive, recognizing social policies as essential contributors to healthcare, improved funding structure to broaden the scope of research and scientific studies [91] and enhanced advocacy on public health concerns.

#### 1.9.1. Urban Policies and Stressors

A study aimed at exploring potential patterns between historical discriminatory practices such as redlining and urban wellbeing in selected metropolitan regions in the US identified correlations between the incidence of cancer, asthma, poor mental health and people without health insurance in zones that had previously been redlined [92]. Redlining was one of the several practices that resulted in an unequal system of credit access, hence fostering residential and economic discrimination [93]. Although the practice was outlawed in the United States as part of the Fair Housing Act of 1968, the bulk of those redlined neighborhoods remain for the low-to-moderate wealth class, suffering from skewed distribution of wealth and an increasing prevalence of chronic diseases [94] which are risk factors of a higher incidence of late stage cancer diagnosis [95].

#### 1.9.2. Work Policies and Stressors

The Occupational Safety and Health Administration is mandated to ensure healthy and safe workplace conditions for employees. The General Duty Clause of the Occupational Safety and Health Act of 1970 proscribes that every employer “shall furnish to each of his employees, employment and a place of employment which are free from recognized hazards that are causing or are likely to cause death or serious physical harm to his employees and comply with occupational safety and health standards promulgated under the Act” [96]. This entails authorizing the implementation of the standards created in compliance with the Act, supporting and motivating states in their efforts to maintain safe and healthy work environments and funding research studies, informational campaigns and continuing education and training programs in workplace safety and health. In the US, the social insurance system, which includes programs funded by earmarked tax revenues and other state programs, is implemented to provide welfare benefits, support individuals, or target demographic groups in satisfying basic needs with the aim of alleviating the impact of social stressors and to bridge the disparity gaps in income levels.

## 2. How Environmental Policy Can Alter Exposure to Stressors and Heavy Metals (Pb, Cd) to Diminish EC Incidence

In the United States, poor cancer outcomes and prognosis are highly correlated with lack of health insurance coverage [97] as there is a low likelihood of uninsured persons having access to timely and efficient cancer interventions. A recent study on socioeconomic status and cancer preventive services opined that the enactment of the Affordable Care Act (ACA) in 2010, which gave previously uninsured or underinsured individuals access to improved health insurance coverage, reduced the economic barriers that created disparities in cancer screening and access to preventive services such as mammography [98], with a significant improvement in early stage at cancer diagnosis [99].

To reduce the rate of exposure to EC risk factors, there must be concerted efforts in ensuring strict implementation of health and environmental standards and regulations. The concentration of heavy metals in various mediums through which they are stored should be regularly monitored [100] with continuous assessment to check bioaccumulation and transfer into food chains. Environmental, economic and social systems should strive to mitigate vulnerabilities and alleviate the consequences of disproportionate income gaps, including past and present institutionalized racism.

## Figures and Tables

**Table 1:** Lead Exposure Policies by Year Enacted and Environmental Medium.

Sources of Lead Exposure				
Regulations	Soil	Air	Water	Paint
Residential Lead-Based Paint Hazard Reduction Act (1992)	X			X
Clean Water Act as amended in 1972	X		X	
Safe Drinking Water Act (1974)			X	
Toxic Substances Control Act (1976)	X		X	X
Clean Air Act (1963)		X		
Comprehensive Environmental Response, Compensation, and Liability Act (1980)	X	X	X	

## Data Availability

Not applicable.
